# Upregulation of NR2B Subunits of NMDA Receptors in the Lateral Parabrachial Nucleus Contributes to Chronic Pancreatitis Pain

**DOI:** 10.1111/cns.70313

**Published:** 2025-02-28

**Authors:** Jing‐Lai Wu, Wen‐Qiong Kuang, Zheng‐Yan Zhu, Jing‐Heng Dou, Jia‐He Yao, Jing Cao, Fu‐Chao Zhang, Guang‐Yin Xu

**Affiliations:** ^1^ Clinical Research Center of Neurological Disease The Second Affiliated Hospital of Soochow University, Jiangsu Key Laboratory of Neuropsychiatric Diseases and Institute of Neuroscience, Soochow University Suzhou China; ^2^ Department of Anatomy School of Basic Medical Sciences, Zhengzhou University Zhengzhou Henan China

**Keywords:** chronic pancreatitis pain, glutamatergic neurons, lateral parabrachial nucleus, NR2B receptors

## Abstract

**Aims:**

Chronic pancreatitis (CP) is a localized or diffuse chronic progressive inflammation of the pancreas that can be caused by a variety of factors and is characterized by abdominal pain. However, the underlying mechanisms are poorly understood. Increasing evidence suggests that central sensitization plays a crucial role in the development of visceral pain, but the precise mechanisms of central neural processing remain unclear.

**Methods:**

CP was induced using repeated intraperitoneal injections of caerulein in mice. Neurospecific anterograde tracing was achieved using herpes simplex virus type 1 (HSV‐1). Fiber photometry was used to assess neuronal activity. Optogenetic, chemogenetic, or pharmacological approaches were applied to manipulate the lateral parabrachial nucleus (LPB) glutamatergic neurons. The abdominal withdrawal threshold (AWT) was measured to evaluate the CP pain. A glutamate sensor was used to detect glutamate release in the LPB.

**Results:**

In the present study, we demonstrated that glutamatergic neurons in the LPB are activated in CP mice, leading to the development of CP pain. Notably, glutamatergic release is increased in the LPB, and the increased release primarily mediates CP pain by binding to the N‐methyl‐D‐aspartate (NMDA) receptor rather than α‐amino‐3‐hydroxy‐5‐methyl‐4‐isoxazolepropionic acid (AMPA) receptors. Specifically, this process involves the binding of the N‐Methyl‐D‐Aspartate Receptor Subunit 2B (NR2B) in the LPB, leading to the development of CP pain.

**Conclusions:**

This study identified the NR2B subunits of NMDA receptors in the LPB as playing a critical role in the regulation of CP pain.

## Graphical abstract

A structural connection between the pancreas and the brain was established and identified the mechanisms through which the lateral parabrachial nucleus (LPB) modulates chronic pancreatitis pain.

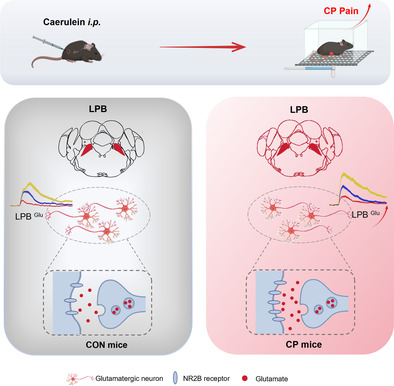

## 1 Introduction

Chronic pancreatitis (CP) is a multifactorial fibrous inflammatory syndrome characterized by recurrent pancreatic inflammation that leads to extensive fibrotic tissue replacement [1]. This condition results in chronic pain, disturbances in both exocrine and endocrine pancreatic function, a reduction in quality of life, and a decreased life expectancy [2, 3]. Currently, most research on the pathogenesis of CP pain has focused on molecular events in the peripheral nervous system and spinal cord [4, 5, 6]. However, studies on the brain are very limited, and the role of the brain in regulating CP pain remains poorly understood.

For pain signaling, peripheral nociceptive stimuli are transmitted to the spinal cord, then further relayed to the brainstem and thalamus [7]. During this process, the parabrachial nucleus (PBN) in the pons acts as a key center, receiving signals from the spinal cord and transmitting them to the thalamus [8]. Furthermore, the PBN is involved in other important sensory processes, such as itch, orofacial affective pain, and aversive emotional behaviors, including aversion learning, avoidance behavior, and anorexic hunger behavior [9]. The lateral parabrachial nucleus (LPB) is a major subnucleus of the PBN [10, 11]. The LPB is primarily associated with pain, taste, smell, and temperature perception [12]. It receives sensory information and relays this to other brain regions [9, 13]. The LPB is closely related to pain modulation due to its primary reception of nociceptive inputs from dorsal spinal cord projection neurons [12]. A recent study found that the LPB plays a key role in modulating gastric pain [14, 15]. However, the role of the LPB in the development and maintenance of CP pain remains unclear.

Glutamate is the predominant neurotransmitter released by excitatory neurons in the central nervous system (CNS). Glutamate receptors can be classified into two main types based on their mechanisms of action: metabotropic glutamate receptors (mGluRs) and ionotropic glutamate receptors (iGluRs). mGluRs are G‐protein coupled receptors (GPCRs) that modulate both the release of glutamate and the postsynaptic effects [16]. The NMDA receptor is a subtype of ionotropic glutamate receptors (iGluRs) and consists of five subunits: NR1, NR2A, NR2B, NR2C, and NR2D. The typical NMDA receptor is a heterotetramer, composed of two NR1 subunits, which bind glycine, and two NR2 subunits, which bind glutamate. The NR2 subunits are randomly selected from the four isoforms, NR2A to NR2D. These receptors are widely distributed throughout the CNS and are critical in excitatory synaptic transmission [17]. Glutamate binding to NMDA receptors leads to the opening of Ca^2+^ channels and induces CNS excitation. Studies have shown that activation of NMDA receptors can induce central sensitization, and upregulation of NMDA receptors can lead to visceral hypersensitivity [18].

This study aims to investigate the neural and molecular mechanisms by which the LPB mediates CP pain in mice. To achieve this, we will employ a range of approaches, including virus‐based tracing and mapping, in vivo neuronal activity recording, and functional modulation of neuronal activity using optogenetics and chemogenetics. Furthermore, this research will contribute to the mapping of the pancreas–brain axis, enhance our understanding of the neural mechanisms underlying CP pain, and provide potential therapeutic strategies for the clinical management of CP‐related pain.

## 2 Materials and Methods

### 2.1 Experimental Animals

Adult male C57BL/6J mice (6–8 weeks old, 20–25 g) were procured from Vital River Corporation and housed at the Experimental Animal Center of Soochow University. Mice were randomly assigned to cages with a maximum of five mice per cage. They were maintained under controlled conditions of temperature (20°C ± 1°C) and humidity, with a 12‐h light/dark cycle and ad libitum access to food and water. All experimental procedures were conducted in accordance with guidelines set forth by the International Association for the Study of Pain (IASP). Ethical approval for the study was obtained from the Ethics Committee for Animal Experimentation at Soochow University.

### 2.2 Chronic Pancreatitis (CP) Model Establishment

The CP model was established by repeated intraperitoneal injections of caerulein (MCE, USA) [19, 20]. Caerulein was purchased from MCE with a specification of 1 mg, and the stock solution was prepared before use. To prepare the stock solution, 1 mg of caerulein was dissolved in 6.67 mL of PBS, resulting in a concentration of 150 mg/mL, and stored at −30°C. Before use, 1 mL of stock solution was diluted with 9 mL to prepare a working solution with a concentration of 15 mg/mL. Mice were anesthetized with isoflurane and measured their body weight. Caerulein was then injected intraperitoneally at a dose of 50 μg/kg, with a frequency of five times daily, each injection spaced 1 h apart, 3 days per week, for 4 weeks. CON mice received an equivalent dose of solvent based on their body weight.

### 2.3 Pancreas‐Brain Virus Tracing

Neurospecific polysynaptic anterograde tracing was performed using type 1 herpes simplex virus (HSV‐1) strain HSV‐tdTomato (BrainVTA, Wuhan, China) along with a titer of 2.0E + 09 PFU/mL. To aid HSV virus infection, the immunosuppressant bortezomib (0.1 mg/mL, Selleckchem, Houston, USA) was administered intraperitoneally 24 h prior to viral injection [21]. Using a microinjector, HSV virus was injected at three different sites in the pancreas: the head, the pancreatic duct, and the tail, with 1 μL of virus per site, totaling 3 μL.

### 2.4 CP Pain Test

Abdominal withdrawal threshold (AWT) was measured to evaluate the CP pain [4, 22, 23]. A brief description is as follows. The mouse was placed on a raised wire mesh floor under a clear plastic box, shaving the fur on the abdomen of the mice. The upper abdomen is stimulated by three different filaments in ascending order of intensity. Abdominal sensitivity of each mouse to mechanical stimuli was determined using von Frey filaments with strengths of 0.02, 0.16, and 1.0 g at 30 min after the final dose of caerulein. The mechanical stimulation with each thread was applied five times at intervals of 5–10 s, and this was repeated five times after a 1 min resting period, for a total of 10 stimulations. To account for “ceiling” effects or desensitization, consecutive stimulations at the same point were avoided. The injury behavior scoring was defined as follows: 0 points for no response; 1 point for an immediate escape or licking/scratching at the site stimulated with von Frey filaments; and 2 points for a strong abdominal contraction or jumping. Data were presented as the total score of responses elicited by 10 challenges with each filament.

### 2.5 Immunofluorescence Staining

Mice were deeply anesthetized with isoflurane and intracardially perfused with 0.9% saline, followed by 4% paraformaldehyde (PFA). Brain tissue was collected and fixed in 4% PFA at 4°C for 6 h, followed by gradient dehydration with 20% and 30% sucrose. Coronal brain sections (30 μm) were cut using a Cryostat microtome (Leica, CM1950) at −25°C. The sections were washed three times with PBS and blocked with a blocking solution (7% normal donkey serum, 0.3% Triton X‐100, and 0.05% sodium azide) for 1 h at room temperature. The sections were then incubated overnight at 4°C with the appropriate primary antibodies, diluted in blocking buffer. The primary antibodies used were: mouse anti‐c‐Fos (Santa Cruz, sc‐271,243, 1:400), rabbit anti‐c‐Fos (Cell Signaling, 2250S, 1:400), rabbit anti‐GABA (Sigma, A2052, 1:400), and rabbit anti‐glutamate (Sigma, G6642, 1:400). After three washes with PBS, the sections were incubated with the appropriate secondary antibodies for 1 h at room temperature. The secondary antibodies used were: Donkey anti‐rabbit Alexa Fluor 488 (Invitrogen, A21206, 1:300), Donkey anti‐mouse Alexa Fluor 555 (Invitrogen, A31507, 1:300), Donkey anti‐mouse Alexa Fluor 488 (Invitrogen, A31507, 1:300), and Donkey anti‐rabbit Alexa Fluor 555 (Invitrogen, A31572, 1:300). Following three additional washes with PBS, the sections were mounted in a medium containing 40,6‐diamidino‐2‐phenylindole, dihydrochloride (DAPI, Abcam, AB104139).

### 2.6 Stereotaxic Injection and Optical Fiber Implantation

Mice were deeply anesthetized using isoflurane, and a stereotactic frame (RWD 71,000‐M, Shenzhen, China) was used for stereotaxic brain injection. The pipette was left in place for 10 min post‐infusion to prevent viral overflow. Coordinates were determined using anterior–posterior (AP) distance from bregma, medial‐lateral (ML) distance from the midline, and dorsal‐ventral (DV) distance from the brain surface. Virus suspension was loaded into a 10 μL syringe (Gaoge, Shanghai, China) connected to a glass micropipette with a tip. A total of 250 nL was injected at a rate of 30 nL/min into the target regions (LPB: AP = −5.3 mm, ML = 1.5 mm, DV = 3.5 mm).

For optic fiber implantation, a fiber optic (200 μm core diameter, ThinkerTech, Nanjing, China) was inserted 100 μm above the injection site and secured with Metabond (Parkell) and dental adhesive. Following behavioral assessments, immunohistochemical analysis was conducted to verify the viral injection, expression, and fiber optic placement.

### 2.7 Fiber Photometry Recordings

Fiber photometry (ThinkerTech, Nanjing, China) was used to record neuronal calcium activity in the LPB following viral transfection with a calcium indicator. A low‐autofluorescence, 200‐μm core, 0.37‐NA optical fiber (ThinkerTech, Nanjing, China) was implanted 0–100 μm above the virus injection site and affixed to the skull using dental cement. Fluorescence signals were acquired at a sampling frequency of 100 Hz through a data acquisition system, including an amplifier (C7319, Hamamatsu) and a photomultiplier tube. Data were classified according to behavioral events in individual experiments, and each stimulus was repeated nine times to ensure the accuracy and reliability of the results. Fiber‐optic recording data were analyzed using custom‐written MATLAB code, with ΔF/F measured 2 s before stimulation as the baseline. The ΔF/F value was calculated as (F‐F0)/F0, where F represents the fluorescence intensity during the event and F0 represents the baseline fluorescence intensity. Experimental data were analyzed and visualized using GraphPad Prism 8 software (San Diego, CA, USA) and the ThinkerTech fiber photometric analysis software package.

### 2.8 Western Blotting

The LPB sample was homogenized in tissue lysate according to the weight of the tissue [24, 25]. Ultrasonic homogenization (PW = 5.0 W) was performed in an ice‐water mixture until the sample became transparent. Following homogenization, the samples were placed on ice for 2 h, then centrifuged at 15,000 rpm for 30 min at 4°C to collect the supernatant. Protein concentration was determined using a BCA protein assay kit (Beyotime, China). Equal amounts of protein (30 μg) were separated by 4% and 10% SDS‐polyacrylamide gel electrophoresis (PAGE), and the protein bands were transferred to a nitrocellulose membrane (Merck Millipore, Germany). The membrane was incubated overnight at 4°C with primary antibodies (1:2000, anti‐GAPDH; 1:2000, anti‐NR2B; 1:2000, anti‐NR2A; 1:2000, anti‐NR2C; 1:2000, anti‐NR2D; Sigma). After washing with TBST, the membrane was incubated with goat anti‐mouse IgG. The proteins were detected using enhanced chemiluminescence (ECL, NCM Biotech, China) and visualized with an appropriate imaging system (Bio‐Rad). The gray values of the protein bands were quantified using ImageJ software. Band intensities were normalized to GAPDH as a reference and statistically analyzed.

### 2.9 Statistical Analyses

All data are statistically analyzed and graphed using GraphPad Prism 8 software. Results are presented as means ± standard error of the mean (SEM), with “n” representing the number of animals. The normality of data distribution was assessed using the Kolmogorov–Smirnov test. Data that do not exhibit a normal distribution were analyzed using the Mann–Whitney test. Unless otherwise indicated, comparisons between two groups were made using the Student's *t*‐test, and comparisons among multiple groups were performed using two‐way ANOVA. A p‐value of < 0.05 was considered statistically significant.

## 3 Results

### 3.1 Identification of a Connectivity Pathway From the Pancreas to the LPB

**FIGURE 1:**
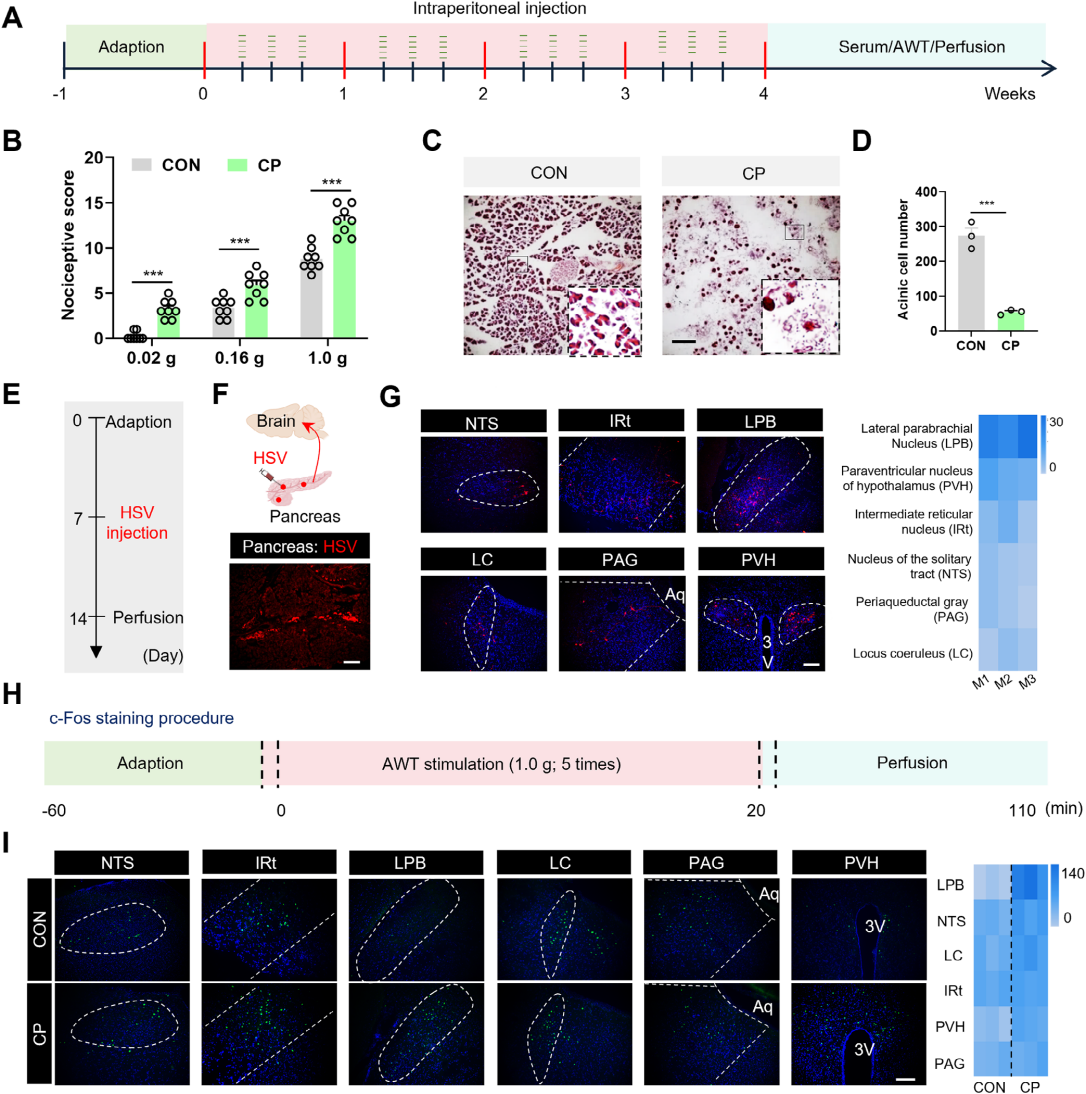
The LPB has structural and functional connections with the pancreas. (A) The diagram of the CP mouse model establishment. (B) AWT score histogram of mice stimulated by 0.02 g, 0.16 g and 1.0 g von Frey fiber (*n* = 8 mice for each group, ***p < 0.001, two‐way ANOVA followed by Sidak's multiple comparison test). (C) Representative pancreatic HE stains image. Scale bar = 50 μm. (D) The graph of pancreatic acinar cells number (*n* = 3 mice for each group, ***p < 0.001, Student's *t*‐test). (E) Timeline diagram of HSV virus injection. (F) Schematic representation of HSV virus injection sites and expression in the pancreas. Scale bar = 50 μm. (G) Red viral fluorescent markers are observed in the central NTS, IRT, LPB, LC, PAG, and PVH regions after injection of HSV‐tdTomato virus into the pancreas. (H) Schematic of c‐Fos expression evoked by von Frey stimulation. (I) Representative maps and thermograms of c‐Fos staining in mice of CON and CP groups (*n* = 3 mice for each group, *p < 0.05, **p < 0.01, ***p < 0.001, Student's *t*‐test). n.s. indicated nonsignificant differences, *p* > 0.05. ANOVA, analysis of variance.

The CP model was established using repeated intraperitoneal injections of caerulein in mice (Figure 1A). Measurement of abdominal withdrawal threshold (AWT) revealed that CP mice exhibited significant pain responses compared to the control (CON) mice (Figure 1B). Hematoxylin and eosin (HE) staining results indicated that the pancreases of CP mice showed pronounced inflammatory responses, with acinar cell atrophy and a significant reduction in cell numbers after caerulein injection (Figure 1C,D). These results suggest that the modeling was successful and that the mice developed CP pain.

To establish structural connections between the pancreas and the CNS, we injected an anterogradely transfected, polysynaptic type 1 herpes simplex virus (HSV‐1) H129 strain expressing red fluorescent protein (HSV‐tdTomato) into the pancreas (Figure 1E,F). To enhance HSV expression, mice were pretreated with immunosuppressants. After 7 days of viral expression, we observed fluorescent labeling in the nuclei of the lateral parabrachial nucleus (LPB), solitary tract (NTS), intermediate reticular nucleus (IRT), locus coeruleus (LC), periaqueductal gray (PAG), and paraventricular hypothalamic nucleus (PVH), indicating anatomical projections between these nuclei and the pancreas (Figure 1G).

Subsequently, we investigated the expression of c‐Fos in the HSV‐labeled brain regions in CON and CP mice using immunofluorescence staining (Figure 1H). Compared to control mice, CP mice exhibited a significantly increased number of c‐Fos‐positive cells in the LPB, NTS, and PVH. Statistical analysis revealed that the LPB showed the most pronounced increase in c‐Fos‐positive cells (Figure 1I). These results collectively demonstrate the identification of the neural pathway connecting the LPB and the pancreas through both structural and functional analyses.

### 3.2 Abdominal Stimulation Significantly Enhances the Calcium Activities of the LPB Glutamatergic Neurons

**FIGURE 2:**
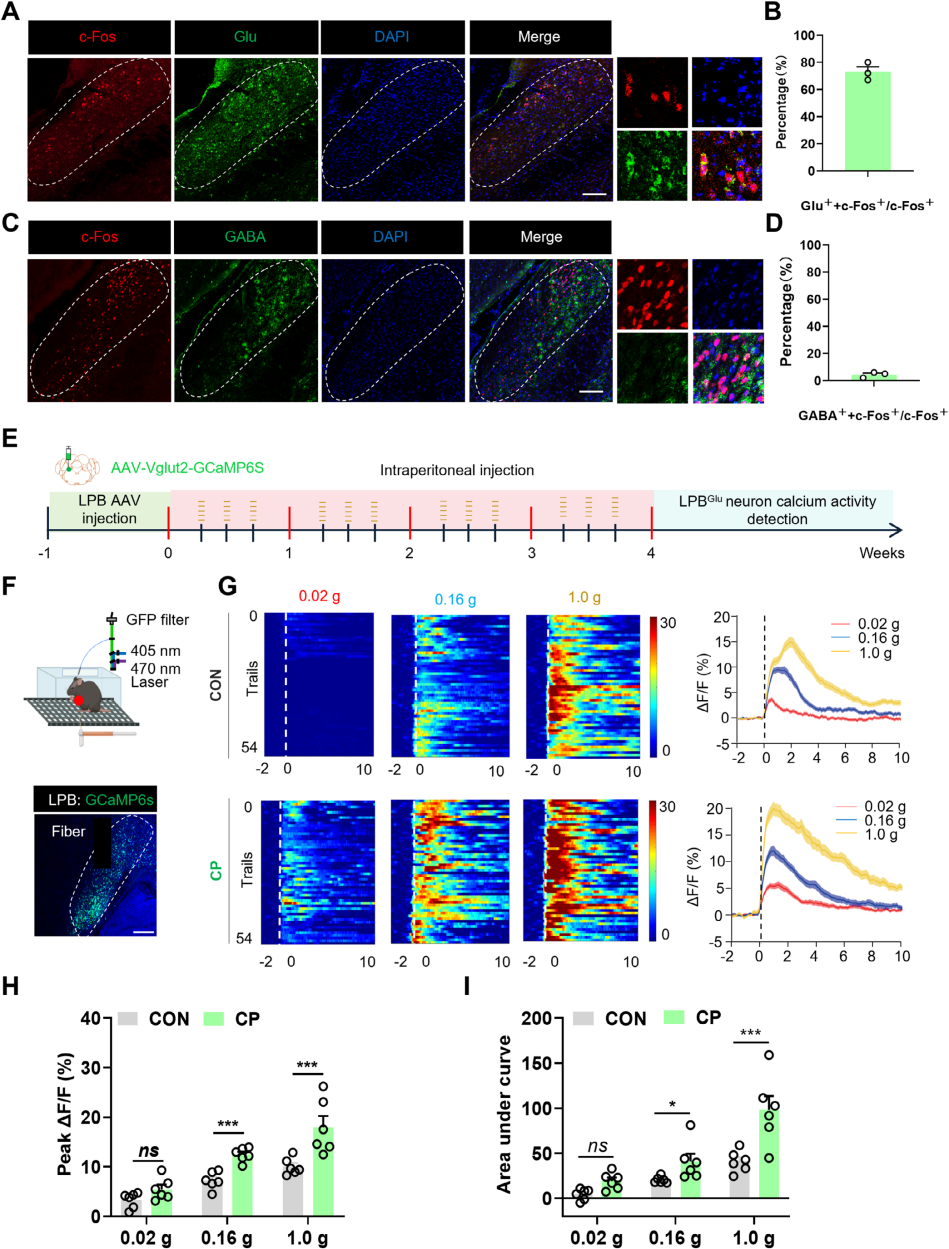
Abdominal stimulation increases the calcium activities of the LPB glutamate neurons. (A) Representative images of c‐Fos^+^ cells (red), glutamatergic neurons (green) and DAPI (blue) co‐expressed in the LPB. Scale bar = 50 μm. (B) Percentage of glutamatergic c‐Fos + neurons (*n* = 3 mice for each group). (C) Representative images of c‐Fos^+^ cells (red), GABAergic neurons (green) and DAPI (blue) co‐expressed in the LPB. Scale bar = 50 μm. (D) Percentage of GABAergic c‐Fos^+^ neurons (*n* = 3 mice for each group). (E) Schematic of fiber‐optic calcium signal recording. (F) Representative images of GCaMP6s viral expression sites in LPB, Scale bar = 50 μm. (G) Heat maps of calcium transient changes in LPB of CON and CP mice during abdominal stimulation using von Frey. (H) Statistical analysis of the average peak ΔF/F (top) of calcium ion activity in glutamatergic neurons in the LPB of CON and CP mice subjected to von Frey abdominal stimulation (*n* = 6 mice for each group, ***p < 0.001, two‐way ANOVA followed by Sidak's multiple comparison test). (I) Statistical analysis of the area under curve (bottom) of calcium ion activity in glutamatergic neurons in the LPB of CON and CP mice (*n* = 6 mice for each group, *p < 0.05, ***p < 0.001, two‐way ANOVA followed by Sidak's multiple comparison test). n.s. indicated nonsignificant differences, *p* > 0.05. ANOVA, analysis of variance.

The immunofluorescence co‐localization was conducted to validate the neuronal types activated in the LPB in CP mice. The staining results indicated that 76% of the activated c‐Fos positive cells co‐labeled with glutamatergic neurons (Figure 2A,B). Only 2% of c‐Fos positive cells co‐labeled with GABAergic neurons (Figure 2C,D). The data suggested that the c‐Fos positive cells activated in the LPB were primarily glutamatergic neurons in the CP mice. To further assess neuronal excitability in the LPB of CON and CP mice during abdominal stimulation, a fiber‐optic calcium imaging system was employed to monitor real‐time changes in calcium signals of glutamatergic neurons in the LPB (Figure 2E). The calcium indicator virus AAV‐Vglut2‐GCaMP6s, which specifically infected glutamatergic neurons, was injected into the LPB of CON and CP mice separately (Figure 2F). CP was induced by injecting caerulein for 4 weeks during viral expression in mice. CON and CP mice underwent varying intensity von Frey filament stimulation on their abdomens, and the real‐time calcium activity of the LPB glutamatergic neurons was recorded. The results revealed that under 0.16 g and 1.0 g filament stimulation, the area under the curve (AUC) of calcium activity and the peak ΔF/F of calcium ion activity in the LPB glutamatergic neurons of CP mice was significantly higher than that of CON mice (Figure 2G). Statistical analysis of the AUC and peak values showed significant differences compared to the CON group (Figure 2H,I). These data showed that von Frey filament stimulation significantly enhanced the excitability of the LPB glutamatergic neurons in CP mice, suggesting that the LPB glutamatergic neurons were activated by the caerulein injection.

### 3.3 The LPB Glutamatergic Neurons Modulated CP Pain Behaviors

**FIGURE 3:**
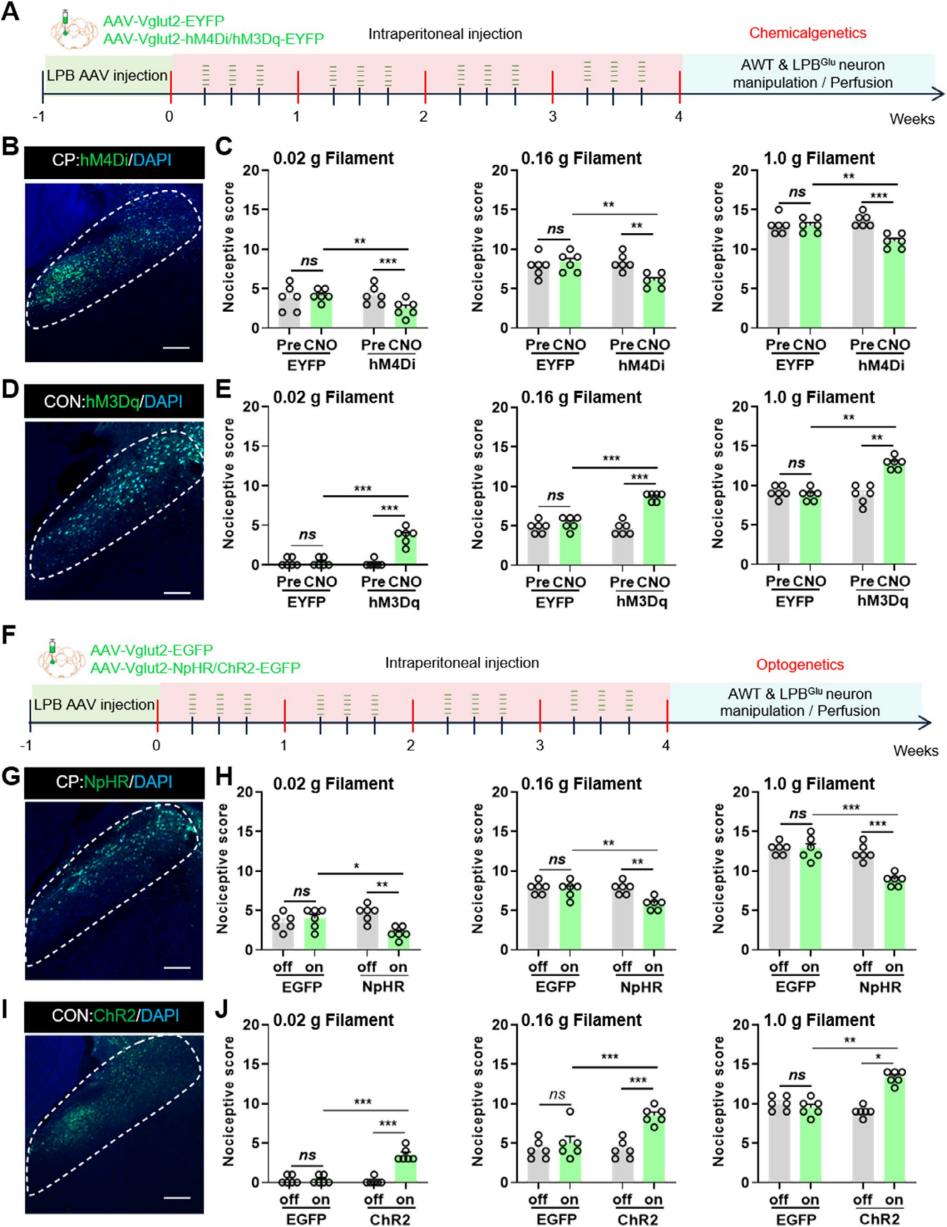
The LPB glutamate neurons modulate CP pain behavior. (A) Timeline of chemogenetic virus injection in mice. (B) Representative image of hM4Di virus expression in the LPB in CP mice. Scale bar = 50 μm. (C) AWT scores of CP mice injected with hM4Di/EGFP virus under 0.02 g, 0.16 g, and 1.0 g stimuli (*n* = 6 mice for each group, **p < 0.01, ***p < 0.001, two‐way ANOVA followed by Sidak's multiple comparison test). (D) Representative image of hM3Dq virus expression in the LPB in CON mice. Scale bar = 50 μm. (E) AWT scores of CON group mice injected with hM3Dq/EGFP virus under 0.02 g, 0.16 g, and 1.0 g stimuli (*n* = 6 mice for each group, **p < 0.01, ***p < 0.001, two‐way ANOVA followed by Sidak's multiple comparison test). (F) Timeline of optogenetic virus injection in mice. (G) Representative image of NpHR virus expression in the LPB in CP mice. Scale bar = 50 μm. (H) AWT scores of CP mice injected with NpHR/EGFP virus under 0.02 g, 0.16 g, and 1.0 g stimuli (*n* = 6 mice for each group, *p < 0.05, **p < 0.01, ***p < 0.001, two‐way ANOVA followed by Sidak's multiple comparison test). (I) Representative image of ChR2 virus expression in the LPB in CON mice. Scale bar = 50 μm. (J) AWT scores of CP mice injected with ChR2/EGFP virus under 0.02 g, 0.16 g, and 1.0 g stimuli (*n* = 6 mice for each group, *p < 0.05, **p < 0.01, ***p < 0.001, two‐way ANOVA followed by Sidak's multiple comparison test). n.s. indicated nonsignificant differences, *p* > 0.05. ANOVA, analysis of variance.

The role of LPB glutamatergic neurons in CP pain was further explored using both chemogenetic and optogenetic techniques. In the chemogenetic approach, AAV2/9‐Vglut2‐hM4Di‐EGFP or AAV2/9‐Vglut2‐EGFP viruses were injected into the LPB (Figure 3A,B). After CNO administration, AWT testing was conducted 40 min later. The results showed that inhibiting LPB glutamatergic neurons in CP mice significantly reduced AWT scores, while no change was observed in the control group (Figure 3C). In the CON group, activation of LPB glutamatergic neurons using AAV2/9‐Vglut2‐hM3Dq‐EYFP and CNO administration led to a significant increase in AWT scores, whereas control virus‐injected mice showed no change (Figure 3D,E). To further validate these findings, optogenetic experiments were conducted. CP mice received injections of AAV2/9‐Vglut2‐NpHR‐EGFP or AAV2/9‐Vglut2‐EGFP viruses, and CP pain was assessed using AWT (Figure 3F). CON group mice were injected with AAV2/9‐Vglut2‐ChR2‐EGFP or AAV2/9‐Vglut2‐EGFP. Von Frey stimulation at 0.02 g, 0.16 g, and 1.0 g was applied to the mice's abdomen, and light‐on/off responses were recorded. Yellow and blue light were used to selectively inhibit or activate LPB glutamatergic neurons, respectively. The results showed that yellow light‐induced inhibition of LPB glutamatergic neurons significantly decreased AWT scores in CP mice (Figure 3G,H), while blue light‐induced activation of these neurons significantly increased AWT scores in CON group mice. No change in AWT scores was observed in the control virus group (Figure 3I,J). These findings confirmed our hypothesis that inhibiting LPB glutamatergic neurons alleviates CP pain in mice, while activating these neurons induces CP pain in the CON group.

### 3.4 NMDA Receptor Antagonist MK‐801 Relieved Pain in CP Mice

**FIGURE 4:**
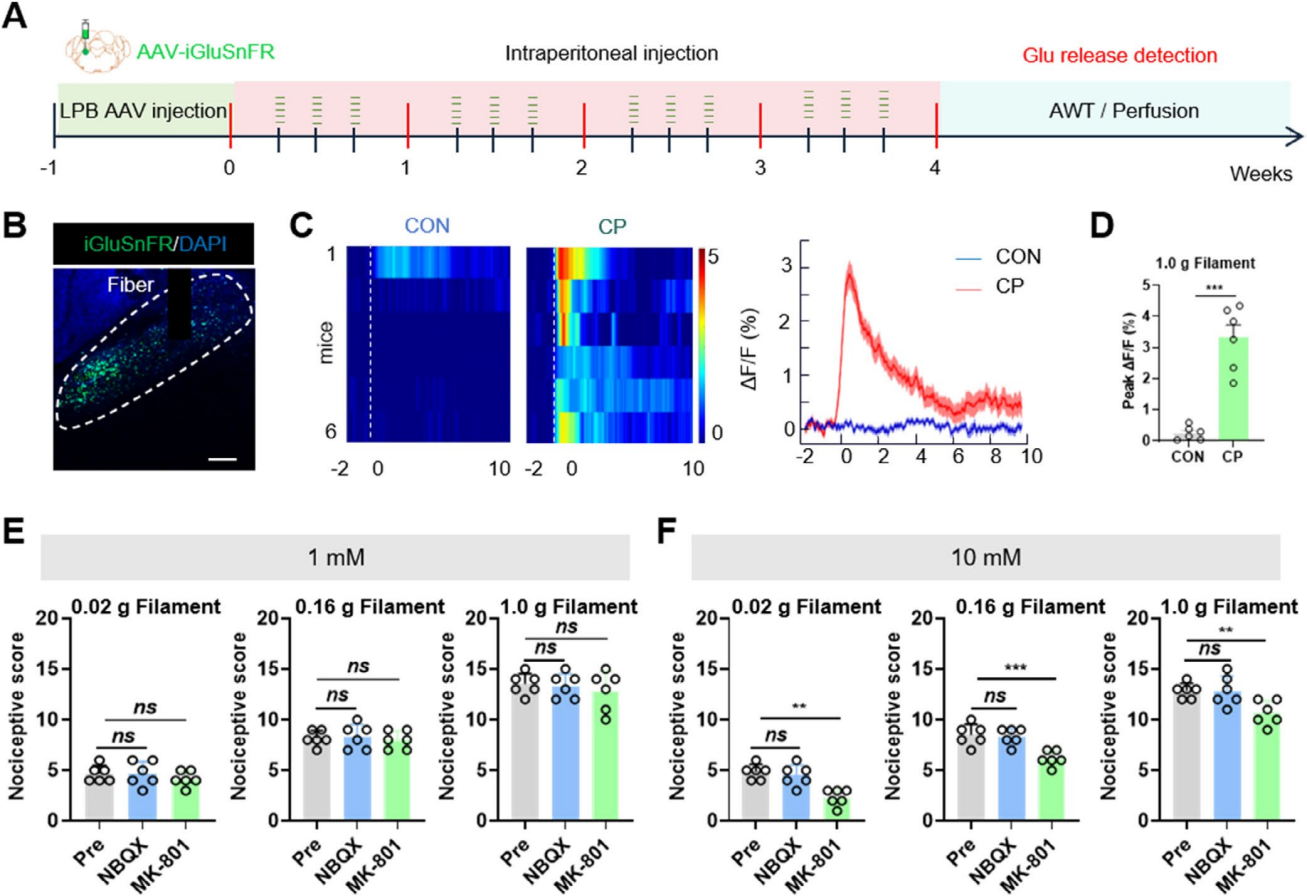
The NMDA receptors antagonist MK‐801 relieves CP pain in mice. (A) The timeline for the viral delivery of a glutamate probe in the LPB. (B) Representative image of iGluSnFR expression in the LPB. Scale bar = 50 μm. (C) Thermogram and peak representation of glutamate release from LPB in CON and CP mice. (D) Statistical analysis of average peak ΔF/F for iGluSnFR activity in the LPB (*n* = 6 mice for each group, ***p < 0.001, Student's *t*‐test). (E) Statistical analysis of AWT scores in CP mice after administering 1 mM MK‐801 and NBQX to the LPB via cannula (*n* = 6 mice per group, *p* > 0.05, Student's *t*‐test). (F) Statistical analysis of AWT scores in CP mice after administering 10 mM MK‐801 and NBQX via cannula (*n* = 6 mice per group, **p < 0.01, ***p < 0.001, two‐way ANOVA followed by Sidak's multiple comparison test). n.s. indicates nonsignificant differences, *p* > 0.05. ANOVA, analysis of variance.

The LPB was an important center for pain signaling, and the neural network it comprised was highly complex in the brain. The LPB mainly received glutamatergic inputs from the spinal cord or other brain regions during pain states [12, 13]. Therefore, glutamate release in the LPB of mice was examined under CP pain conditions. The levels of glutamate release in the LPB were measured following 1.0 g von Frey fiber stimulation of the mouse abdomen (Figure 4A,B). The results showed a significant increase in glutamate release in the LPB of CP mice compared to CON mice following the 1.0 g von Frey fiber stimulation (Figure 4C,D). After confirming the increase in glutamate release in the LPB, we identified the glutamate receptors involved. Both NMDA and AMPA receptors, which are ionotropic glutamate receptors, were found to be responsible for rapid excitatory synaptic transmission in the CNS [26]. We implanted cannulas in the LPB and, using pharmacological approaches, injected the NMDA receptor antagonist MK‐801 and the AMPA receptor antagonist NBQX through these cannulas [27, 28, 29]. When 1 mM concentrations of NBQX and MK‐801 were administered to the mice, the CP pain responses remained unchanged (Figure 4E). Upon increasing the antagonist concentrations to 10 mM, we observed that the AWT score of CP mice significantly decreased following MK‐801 administration, while NBQX had no effect on the CP pain behavior of the mice (Figure 4F). These results indicated that glutamate released into the LPB primarily bound to NMDA receptors, and the NMDA receptor‐specific antagonist MK‐801 significantly alleviated CP pain responses in mice.

### 3.5 The NR2B Subtypes of the LPB NMDA Receptors Modulated CP Pain in Mice

**FIGURE 5:**
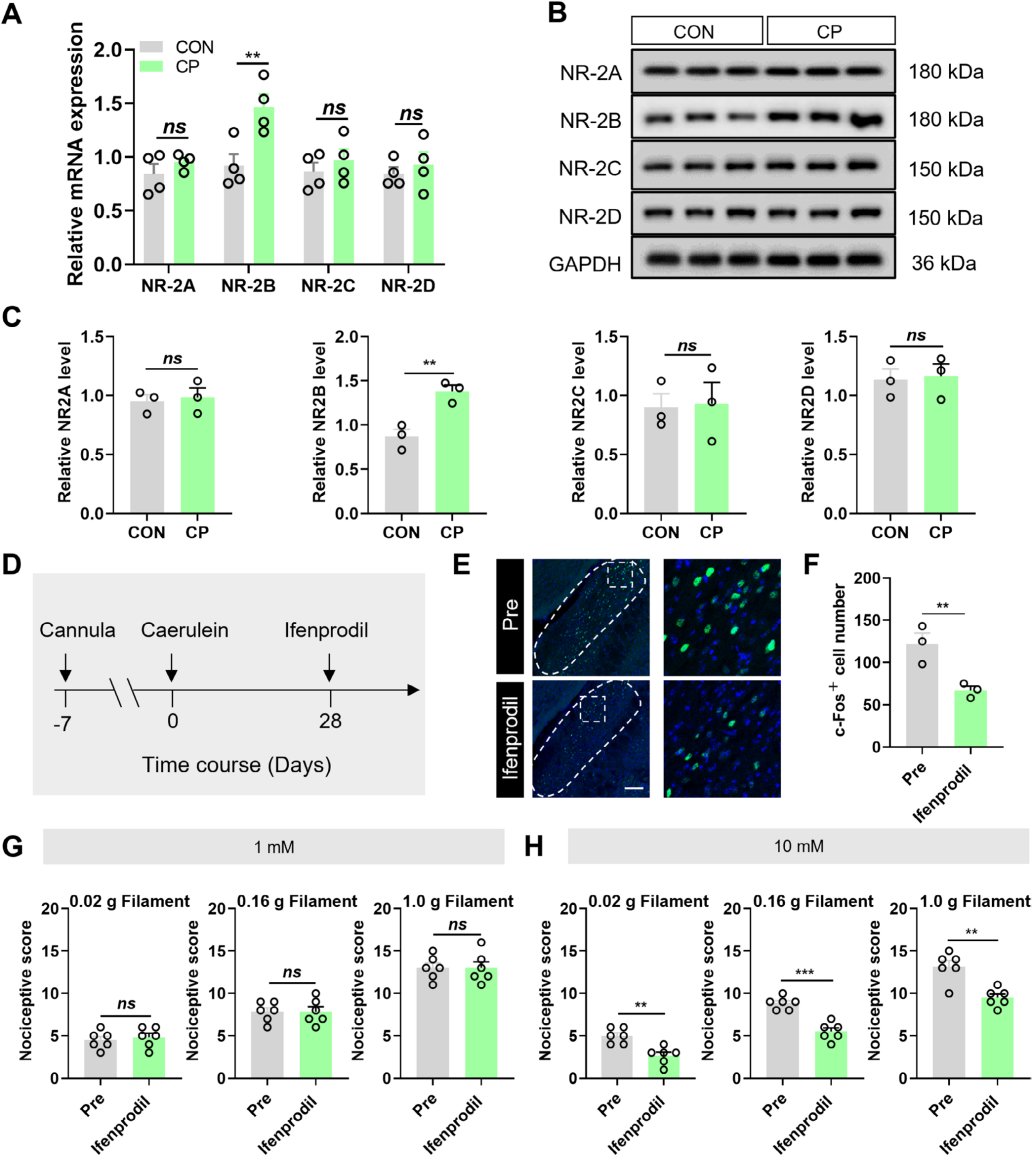
The NR2B subunits of the LPB NMDA receptors modulate CP pain in mice. (A) Bar graph of NMDA receptor mRNA expression levels in the LPB (*n* = 4 mice per group, **p < 0.01, two‐way ANOVA followed by Sidak's multiple comparison test). (B) Schematic representation of protein level expression of NMDA receptor subtypes in the LPB of CON and CP mice. (C) Bar graph of NMDA receptor protein expression in the LPB (*n* = 3 mice for each group, **p < 0.01, Student's *t*‐test). (D) Timeline of NR2B antagonist injection into the LPB. (E) Schematic representation of c‐Fos expression after injection of 10 mM concentration of NR2B receptor antagonist in the LPB of CP mice. Scale bar = 50 μm. (F) Statistical graph of c‐Fos expression in the LPB following antagonist injection (*n* = 3 mice for each group, **p < 0.01, Student's *t*‐test). (G) Pain detection statistics before and after injection of 1 mM antagonist in CP mice (*n* = 6 mice for each group, *p* > 0.05, Student's *t*‐test). (H) Pain detection statistics before and after injection of 10 mM antagonist in CP mice (*n* = 6 mice for each group, **p < 0.01, ***p < 0.001, Student's *t*‐test). n.s. indicated nonsignificant differences, *p* > 0.05. ANOVA represented analysis of variance.

NMDA contains various subunits, and we have further explored its role in the regulation of CP pain. Among the receptor subunits analyzed, the mRNA levels of NR2B receptors in the LPB were significantly elevated (Figure 5A). This finding was confirmed by Western Blot (Figure 5B,C). All of these results indicated that both mRNA and protein levels of NR2B receptors in the LPB were significantly elevated in CP mice. Next, a cannula was implanted in the LPB, and NR2B antagonists ifenprodil were administered through the cannula (Figure 5D). A significant reduction in the number of c‐Fos positive cells occurred after the injection of the NR2B receptor antagonist at a concentration of 10 mM in the LPB of CP mice (Figure 5E,F). After administering 300 μL of a 1 mM concentration of the antagonist, there was no change in pain response in CP mice (Figure 5G). However, after administering a 10 mM concentration of the antagonist, the pain response in CP mice was significantly reduced (Figure 5H). These results suggested that NR2B mediated the glutamatergic neuronal excitability in the LPB of CP mice, leading to altered pain behavior.

## 4 Discussion

**FIGURE 6:**
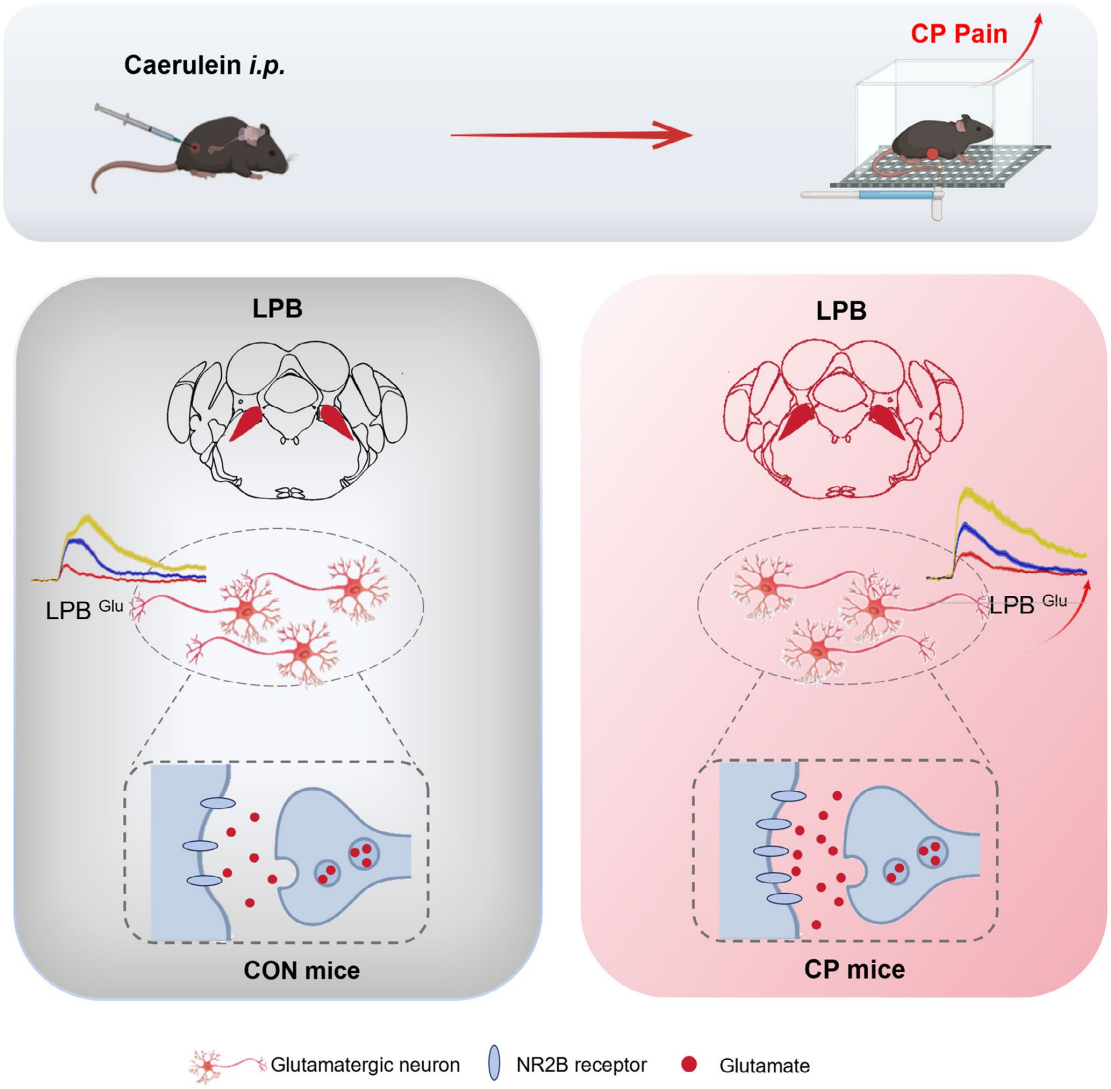
A working model. The LPB glutamatergic neurons were activated in CP mice, leading to the development of CP pain. Glutamate release was increased in the LPB, and the increased release primarily mediates CP pain by binding to NMDA receptors rather than AMPA receptors. Specifically, this process involves binding to NR2B receptors in the LPB, leading to CP pain.

CP pain poses a significant challenge to current understanding and treatment strategies. In our study, we established a structural connection between the pancreas and the brain and identified the mechanisms through which the LPB modulates CP pain (Figure 6). We found that glutamatergic neurons in the LPB play a critical role in mediating CP pain. Activation of these neurons exacerbated CP pain, while their inhibition produced an analgesic effect. Additionally, NR2B receptors in the LPB were found to be involved in the modulation of CP pain in mice. These findings suggest that the LPB may act as a key neural hub for the regulation of CP pain.

One of the key findings is the identification of the structural connection between the LPB and the pancreas using a neuro‐specific anterograde transsynaptic HSV virus. Compared to previous tracing methods, such as cholera toxin subunit B (CTB) dyes [30], the HSV virus offers greater neuro‐specificity and more stable viral signals. Additionally, we confirmed the functional connectivity between the LPB and pancreas through c‐Fos staining, demonstrating that the LPB can be activated by CP pain. Previous studies have shown that CP pain in rats induced a significant increase in c‐Fos expression in both the NTS and ACC, and that the NTS projections to the ACC neural circuits are involved in CP pain [31, 32]. However, by using an HSV virus capable of transsynaptic tracing, we identified the central brain regions receiving projections from the pancreas and found that the LPB exhibited the most significant increase in c‐Fos expression. Additionally, the LPB is a target for nociceptive projection neurons from the spinal dorsal horn, suggesting that the LPB plays a crucial role in the transmission of pain information from the brainstem to the cortex. Nevertheless, the role of the LPB in the neural circuits mediating CP pain requires further validation.

Glutamate is an important neurotransmitter that plays a crucial role in signal transmission in the CNS, particularly in the process of pain perception and modulation [33, 34, 35]. When external noxious stimuli, such as mechanical injury or temperature changes, trigger pain, peripheral sensory nerve endings release glutamate. These neural signals are transmitted to the CNS via the spinal cord. Glutamate mediates excitatory signaling between neurons in the dorsal horn of the spinal cord, ultimately relaying pain information to the brain [36, 37]. It has been reported that the LPB is more receptive to glutamatergic neurons inputs [13]. Similarly, we observed an increase in glutamate transmitter release in the LPB. However, the specific upstream nuclei responsible for the increased glutamate inputs to the LPB in the CP pain state require further investigation. Research demonstrates that NMDA receptors play a crucial role in neural plasticity. NMDA receptors are predominantly expressed in the postsynaptic, and in the context of pain, they enhance the response of dorsal horn sensory neurons to both noxious and innocuous stimuli [38, 39, 40, 41]. These suggest that NMDA receptors facilitate both hyperalgesia and allodynia. Among the subtypes of NMDA receptors, NR2A and NR2B are more commonly studied in pain research [42]. In a formalin‐induced pain model, Researchers observe upregulation of NMDA receptor subunits NR2A and NR2B in the ACC, correlating with the pain produced [43]. NR2A receptors are typically associated with rapid pain transmission in the CNS, while NR2B receptors play a significant role in the maintenance and enhancement of chronic pain, often linked to increased excitability and pain sensitivity in the nervous system [44]. In our study, we found a significant upregulation of NR2B receptors under the CP pain state. However, the antagonist used in our study does not selectively target NR2B receptors on glutamatergic neurons and also affects NR2B receptors expressed on other neurons. Further validation can be conducted by specifically knocking down NR2B receptors expressed on glutamatergic neurons. Nevertheless, this design may also limit the applicability of our findings in female mice. Therefore, future studies should consider the inclusion of female mice in experiments and explore the impact of sex differences in this field.

To sum up, this study identified neural connections from the pancreas to the LPB. The LPB glutamatergic neurons are essential for processing CP pain signals. These findings reveal ascending pathways in the pancreas, enriching the connotation and extension of the pancreas –brain axis, and may provide an effective strategy for the clinical management of patients with CP pain.

## Author Contributions

J.‐L.W. performed experiments, analyzed data and prepared the manuscript. W.‐Q.K. and Z.‐Y.Z. performed experiments, analyzed data, and prepared the figures. J.‐H.D., J.‐H.Y. and J.C. performed experiments, analyzed data. F.‐C.Z. analyzed data, prepared and revised the manuscript. G.‐Y.X. designed experiments, supervised the experiments, and finalized the manuscript. All the authors have read and approved the paper.

## Ethics Statement

The care and handling of the animals was approved by the Institutional Animal Care and Use Committee of Soochow University and was in accordance with the guidelines of the International Association for the Study of Pain.

## Conflicts of Interest

The authors declare no conflicts of interest.

## Supporting information


Data S1.

## Data Availability

The data that support the findings of this study are available from the corresponding author upon reasonable request.
