# Supplementary material for: Upregulation of NR2B Subunits of NMDA Receptors in the Lateral Parabrachial Nucleus Contributes to Chronic Pancreatitis Pain
Source: CNS Neurosci Ther. 2025 Feb 28;31(3):e70313. doi: 10.1111/cns.70313 (PMC11871393; doi:10.1111/cns.70313)
Supplement: Supplementary file 1 — Data S1. [file CNS-31-e70313-s001.pptx]

## Slide 1
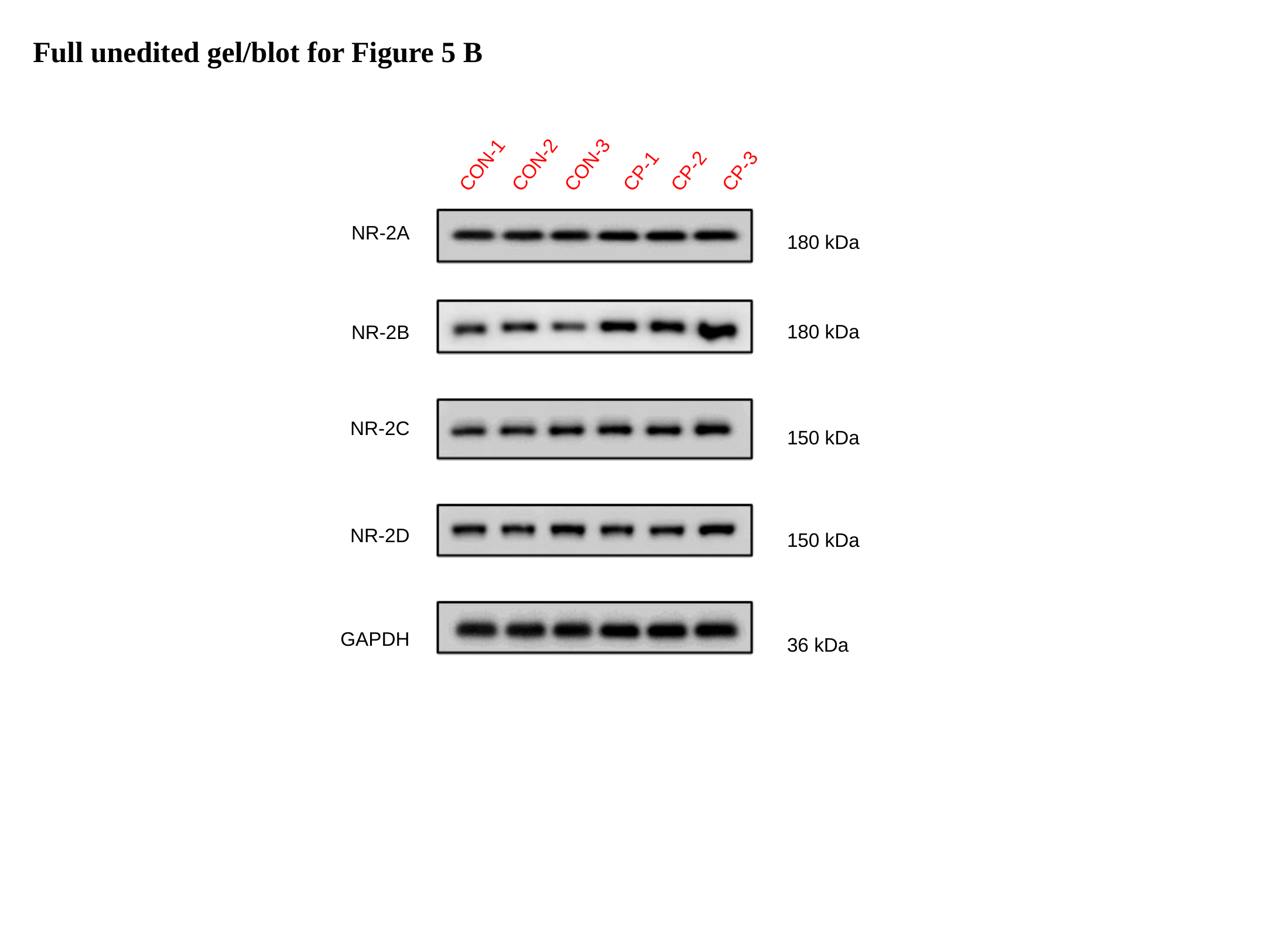

Full unedited gel/blot for Figure 5 B
CON-1
CON-2
CON-3
CP-1
CP-2
CP-3
NR-2A
180 kDa
180 kDa
NR-2B
NR-2C
150 kDa
NR-2D
150 kDa
GAPDH
36 kDa
